# Assessment of US Pharmacies Contracted With Health Care Institutions Under the 340B Drug Pricing Program by Neighborhood Socioeconomic Characteristics

**DOI:** 10.1001/jamahealthforum.2022.1435

**Published:** 2022-06-17

**Authors:** John K. Lin, Pengxiang Li, Jalpa A. Doshi, Sunita M. Desai

**Affiliations:** 1 Department of Medicine, Perelman School of Medicine, University of Pennsylvania, Philadelphia; 2 Leonard Davis Institute of Health Economics, University of Pennsylvania, Philadelphia; 3 Penn Center for Cancer Care Innovation, Abramson Cancer Center, University of Pennsylvania, Philadelphia; 4 Department of Population Health, School of Medicine, New York University, New York

## Abstract

This cross-sectional study assesses pharmacy participation in the 340B Drug Pricing Program following the 2010 expansion and the extent to which growth has occurred in socioeconomically disadvantaged neighborhoods.

## Introduction

The federal 340B Drug Pricing Program entitles participating health care institutions to contract with pharmacies to dispense drugs purchased at discount to their patients. Previously, 340B institutions could contract with only a single pharmacy. In part responding to concerns about patient access in socioeconomically disadvantaged communities,^1^ starting in 2010, 340B institutions could contract with an unlimited number of pharmacies. In this study of annual cross-sections, we measured pharmacy participation following the 2010 expansion and the extent to which growth has occurred in socioeconomically disadvantaged neighborhoods.

## Methods

Using data from the Health Resources and Services Administration, the American Community Survey, and the National Plan and Provider Enumeration System, we constructed a data set comprising all US pharmacies from 2006 to 2019. For each pharmacy, our data included whether the pharmacy contracted with a 340B institution in each year and whether it was a retail, specialty, or mail-order pharmacy.

As socioeconomic measures of the population in each retail pharmacies’ zip code, we used median household income; Social Deprivation Index, a composite measure quantifying socioeconomic disadvantage where higher scores indicate greater social deprivation^2^; racial and ethnic composition, categorized as Black non-Hispanic, Hispanic/Latino, White non-Hispanic, or other (not a majority of Black non-Hispanic, Hispanic/Latino, or White non-Hispanic residents), on the basis of whether greater than 50% of residents self-reported as such^3^; and rural and urban classification.

We measured the number and percentage of pharmacies contracting with at least one 340B institution annually from 2006 to 2019, overall and by pharmacy type. Next, we assessed changes in the distribution of 340B and non-340B retail pharmacies across strata for each socioeconomic characteristic from 2011 (the first full year after the 2010 expansion) to 2019. We then estimated the differential change during this time period between 340B and non-340B pharmacies.^4^ The University of Pennsylvania institutional review board deemed this study exempt because of its policy on nonhuman participant research. We followed the Strengthening the Reporting of Observational Studies in Epidemiology (STROBE) reporting guideline. Data were analyzed using R statistical software, version 4.2.0 (R Project for Statistical Computing).

## Results

A total of 91 740 US pharmacies were assessed from 2006 to 2019. The percentage of pharmacies contracting with a 340B institution pre-expansion (2006-2009) grew minimally (1.2% to 1.7%) (Figure). Postexpansion (2011-2019) growth accelerated overall (5.9% to 29.9%) and among retail (5.7% to 32.6%), specialty (5.3% to 24.7%), and mail-order (4.5% to 20.5%) pharmacies. In 2019, retail pharmacies comprised 92% of 340B pharmacies.

**Figure.**
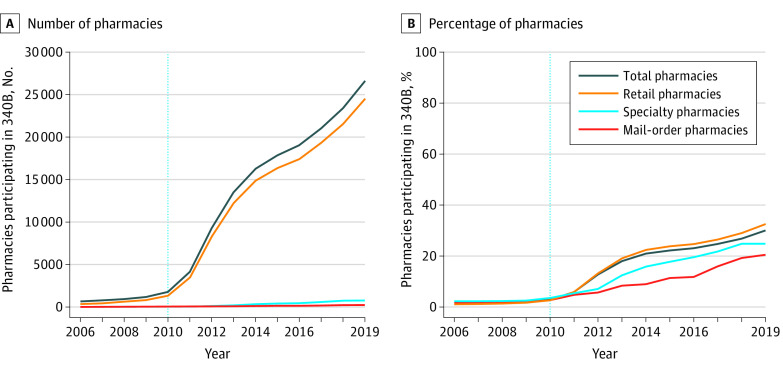
Number and Percentage of Pharmacies Contracting With a 340B Health Care Institution From 2006 to 2019, Overall and by Type of Pharmacy A, The number of US pharmacies contracting with a 340B health care institution by pharmacy type. B, The percentage of US pharmacies contracting with a 340B health care institution by pharmacy type. The dotted vertical lines divide the time series prior to and after the Affordable Care Act, which starting April 2010, allowed 340B health care institutions to contract with an unlimited number of pharmacies. Data on 340B contract pharmacies are from the 340B Office of Pharmacy Affairs database. Data on US pharmacies are from the National Plan and Provider Enumeration System National Provider Identifier registry.

From 2011 to 2019, the share of 340B retail pharmacies in socioeconomically disadvantaged and primarily non-Hispanic Black and Hispanic/Latino neighborhoods declined, even though the share of all retail pharmacies (ie, 340B and non-340B) in socioeconomically disadvantaged and racial and ethnic minoritized neighborhoods increased slightly (Table).

**Table.** Comparison of the Change in Neighborhood Socioeconomic Characteristics for 340B vs Non-340B Retail Pharmacies From 2011 to 2019^a^

Characteristic	Pharmacies, %									Differential change for 340B vs non-340B retail pharmacies from 2011-2019^b^
	All retail			340B retail			Non-340B retail			
	2011	2019	Change	2011	2019	Change	2011	2019	Change	Mean % (95% CI)^c^
Total pharmacies, No.	60 996	75 508	14 512	3493	24 582	21 098	57 503	50 926	−6577	NA
Median family income, $^d^										
<45 000	17.4	18.1	0.7	23.1	17.5	−5.6	17.1	18.4	1.3	−7.0 (−8.6 to −5.4)
45 000-59 999	27.6	27.5	−0.1	32.8	31.3	−1.5	27.2	25.7	−1.6	0.1 (−1.7 to 1.9)
60 000-79 999	27.4	26.9	−0.5	26.2	28.2	2.0	27.5	26.2	−1.2	3.3 (1.5 to 5.0)
≥80 000	27.6	27.4	−0.1	17.9	23.0	5.0	28.1	29.6	1.5	3.6 (2.1 to 5.1)
Social Deprivation Index^e^										
≥60	42.8	43.8	1.0	54.6	47.0	−7.6	42.1	42.3	0.2	−7.8 (−9.7 to −5.9)
37-59	23.9	23.4	−0.4	23.2	24.8	1.6	23.9	22.8	−1.1	2.7 (1.1 to 4.3)
18-36	18.8	18.2	−0.5	13.9	17.0	3.1	19.0	18.8	−0.2	3.4 (2.0 to 4.7)
<18	14.6	14.5	−0.1	8.3	11.2	2.9	14.9	16.1	1.2	1.7 (0.6 to 2.8)
Race and ethnicity^d^										
Black, non-Hispanic/Latino	5.4	5.6	0.2	9.1	5.9	−3.2	5.2	5.5	0.3	−3.6 (−4.6 to −2.5)
Hispanic/Latino	8.9	9.5	0.6	8.7	8.1	−0.6	8.9	10.1	1.3	−1.9 (−2.9 to −0.8)
Other^f^	13.6	14.1	0.5	13.4	13.2	−0.2	13.6	14.6	0.9	−1.1 (−2.4 to 0.2)
White, non-Hispanic/Latino	72.1	70.8	−1.4	68.8	72.8	4.0	72.3	69.8	−2.5	6.5 (4.9 to 8.2)
Geography^g^										
Rural	8.6	8.5	−0.1	13.1	11.6	−1.5	8.3	7.0	−1.3	−0.3 (−1.5 to 1.0)
Urban	91.4	91.5	0.1	86.9	88.4	1.5	91.6	92.9	1.3	0.3 (−1.0 to 1.5)

Abbreviation: NA, not applicable.

^a^ Although the growth in 340B retail pharmacy participation was more concentrated in the most affluent zip codes, the share of 340B pharmacies in the most socioeconomically disadvantaged zip codes declined. This contrasts with non-340B retail pharmacies, which saw relatively little change in this same period. Values are rounded to the nearest tenth of a percent.

^b^ Calculated as the difference between the change in 340B retail pharmacies from 2011 to 2019 and the change in non-340B retail pharmacies from 2011 to 2019.

^c^ CIs calculated from 1000 bootstrap samples.

^d^ Median family income as well as race and ethnicity for each neighborhood were indexed to 2015. Results not indexed to 2015 were similar to the main results.

^e^ Cutoffs for Social Deprivation Index are the approximate 25th (18), 50th (36), and 75th (60) percentiles for zip codes in 2015. Higher scores indicate zip codes that face worse deprivation. We use the Social Deprivation Index score calculated from 2011 to 2015.

^f^ Neighborhoods were categorized as Black non-Hispanic, Hispanic/Latino, or White non-Hispanic on the basis of whether greater than 50% of residents self-reported as such. Neighborhoods in which there was not a majority of White non-Hispanic, Black non-Hispanic, or Hispanic/Latino residents were categorized as other.

^g^ Rural and urban classifications were taken from 2010 US Department of Agriculture rural-urban commuting codes.

Although the percentage of 340B pharmacies in the lowest income neighborhoods declined by 5.6%, the percentage of non-340B pharmacies in the same neighborhoods increased by 1.3% (differential change: –7.0% [95% CI, –8.6% to –5.4%]) (Table). In contrast, though the percentage of 340B pharmacies in the highest income neighborhoods increased by 5.0%, the percentage of non-340B pharmacies in the same neighborhoods increased by 1.5% (differential change: +3.6% [95% CI, 2.1% to 5.1%]).

There were similar differential declines in the percentage of 340B and non-340B pharmacies in neighborhoods with the highest levels of social deprivation (–7.8% [95% CI, –9.7% to –5.9%]), predominantly Black neighborhoods (–3.6% [95% CI, –4.6% to –2.5%]), and predominantly Hispanic/Latino neighborhoods (–1.9% [95% CI, –2.9% to –0.8%]). The percentage of 340B and non-340B pharmacies in rural neighborhoods declined at a similar rate (–0.3% [95% CI, –1.5% to 1.0%]).

## Discussion

Substantial growth occurred following the 2010 340B expansion—by 2019, nearly one-third of all pharmacies were contracting with a 340B institution. The vast majority were retail pharmacies.

Contract pharmacy growth was concentrated in affluent and predominantly White neighborhoods, whereas the share of 340B pharmacies in socioeconomically disadvantaged and primarily non-Hispanic Black and Hispanic/Latino neighborhoods declined. Our study was limited in that we could not observe whether 340B discounts were passed to low-income patients. Nonetheless, our work adds to a growing body of evidence questioning the degree to which 340B program growth serves vulnerable communities.^4,5,6^
